# Study on the co-occurrence of multiple health service needs throughout the lifecourse of rural residents in China based on association rules

**DOI:** 10.3389/fpubh.2024.1480894

**Published:** 2024-11-19

**Authors:** Jingjing Jia, Xuejiao Liu, Panpan Ren, Mengyao Chen, Jinglin Xu, Xiang Zhang

**Affiliations:** ^1^School of Medicine and Health Management, Tongji Medical College, Huazhong University of Science and Technology, Wuhan, China; ^2^Research Centre for Rural Health Service, Key Research Institute of Humanities and Social Sciences of Hubei Provincial Department of Education, Wuhan, China

**Keywords:** multiple service bundling, health service needs, lifecourse, association rules, rural residents in China

## Abstract

**Objective:**

To understand the multiple health service needs of rural residents in China and explore the co-occurrence patterns of these needs throughout the entire life course, providing a basis for the formulation and optimization of health service packaging policies.

**Methods:**

This study utilized a stratified random sampling method, resulting in a final sample size of 15,125 individuals. The R statistical software was employed to apply the Apriori algorithm to mine the co-occurrence relationships among multiple health service needs across the life course and to explore the packaging model of these services.

**Results:**

The health service needs rate among rural residents in China is 86.76%, with a multiple health service needs rate of 78.72%. The most needed services are health exercise guidance (17.10%), Traditional Chinese Medicine health care (15.53%), and internet health information services (14.40%). The highest combined health service need is for “exercise guidance need + internet health information need + Traditional Chinese Medicine health care need,” followed by “exercise guidance need + internet information need.” There are significant differences in the content and strength of associations in the co-occurrence structure of multiple health service needs across different age groups. During the life preparation stage, the need for multiple health services is high, with modern medical care and child management having the highest support. In the life protection stage, the focus shifts to preventive health needs, with strong associations among co-occurring needs (the highest support rule being Traditional Chinese Medicine health care + exercise guidance, support = 21.12%). The co-occurrence of medical and preventive health service needs among the older adult is diverse, with the strongest association being between chronic disease management services and rehabilitation services (support = 31.24%).

**Conclusion:**

The multiple health service needs rate among rural residents in China is high, with the greatest needs being for exercise guidance, Traditional Chinese Medicine health care, and internet health information services. There are significant differences in health service needs across different life stages. It is essential to enhance the integration and packaging of health service resources to promote diversity in health services and meet the multiple health service needs of residents throughout their life courses.

## 1 Introduction

Since China’s reform and opening up, profound changes in the social economy, residents’ values, and social structures have resulted in significant shifts in population structure and disease patterns. Health risks for residents have increasingly transitioned from acute infectious diseases to chronic non-communicable diseases, collectively driving the evolution of health service needs (1). Today, health needs extend beyond mere survival “cure” needs, with the emergence of innovative service models such as integrated medical and nursing care and psychological therapy, leading to the development of consumption, developmental, and social health needs (2). Previous research has largely focused on professional health service needs, assessing residents’ health needs through indicators like disease prevalence, types, and severity (3). However, this biomedical perspective often emphasizes biological factors while overlooking the influence of psychological and social dimensions. In the “big health” era, a singular indicator cannot sufficiently capture the overall range, structure, and hierarchy of residents’ health needs (4). Therefore, accurately identifying the primary health service needs of residents and their interrelationships is crucial for effectively integrating non-professional medical health service systems with other systems and providing grassroots health services.

The life course encompasses all stages from conception to the end of life (5), typically divided into infancy, youth, and old age. Research shows that health needs at different life stages often co-occur (6). Life course theory emphasizes the inherent connections between these stages, demonstrating how early lifestyles and experiences can significantly impact health outcomes in adulthood and old age (7). Therefore, accurately identifying priority health needs for each life stage can shift the focus of health efforts from clinical treatment to health promotion (8), ultimately maximizing individual health potential.

In middle- and low-income countries, residents’ health service needs are diverse and complex (9). Research shows that these needs are influenced not only by economic and social conditions but also by individual lifestyles, cultural backgrounds, and health perceptions (10). There is a growing need for basic health services (11), maternal and child health (12), and chronic disease management (13). Furthermore, the introduction of technologies such as the Internet of Things and the emergence of digital health services present new opportunities to improve service accessibility and quality (14). Additionally, there is an increasing need for mental health and social support, reflecting people’s expectations for integrated health services (15). However, existing literature primarily focuses on individual health services, lacking comprehensive studies on multiple health service needs, particularly regarding specific needs and service models across different life stages.

This study contributes by utilizing association rule algorithms to explore the interrelationships among health service needs, addressing the existing research gap regarding multiple health service needs. Additionally, it presents various combinations of health service needs from a life course perspective, aiming to deepen our understanding of the current state of multiple health service needs among rural residents in China. The findings will provide policy recommendations for disease prevention and population health promotion. This approach will assist government decision-making in the bundled provision of health services, ultimately enhancing the health and well-being of rural residents.

## 2 Data and methods

### 2.1 Data sources

This study employs a stratified random sampling method, selecting one province each from China’s eastern, central, and western regions based on their economic development levels. From each province, two counties and two districts are chosen. Within each sample county or district, five streets (or townships) are selected, and from each street (township), six communities (or villages) are randomly sampled, with 45 households chosen from each community (village). This results in an initial sample size of 4*5*6*40 = 4,800 households. To ensure both the quantity and quality of the data, and considering sampling error and confidence intervals (*e* = 2.5, *z* = 95%), the total sample size is increased to approximately 5,400 households. After excluding non-responses and incorrect answers, the final valid sample consists of 5,040 households, encompassing a total of 15,125 individuals, yielding a response rate of 93.33%. The questionnaire includes sections on basic demographic information, health status, health literacy, utilization of medical services, additional health needs (including requests for service processes and methods, as well as grassroots health services that are expected but not yet provided), and satisfaction levels regarding these needs. To ensure that responses accurately reflect individual viewpoints, guardians provide answers for children aged 0 to 15, and the questionnaire does not ask adolescents about health service needs beyond objective medical necessities.

### 2.2 Health service needs

Basic medical and public health service projects have evolved over time, reflecting the fundamental health needs of residents in different eras. This study is based on the basic medical services and the “14 + 16” basic public health services implemented in China over the past five years (16), along with health need assessment tools (HNAT) to preliminarily design a collection of essential health service projects (17). After manual screening, 12 health service needs are identified, encompassing various fields such as modern medical care, preventive health, and chronic disease management. These include physical exercise guidance, traditional Chinese medicine health care, internet medical care and information, health guidance, chronic disease management, rehabilitation, modern medical care (inpatient and outpatient), child management, pregnancy health management, traditional Chinese medicine consultation needs, mental health care, and home care. The selection criteria are as follows: (1) the services must be perceivable and evaluable by residents; services that cannot be directly perceived, such as community health needs assessments, community health monitoring, and regular reporting, are excluded from this study; (2) the services must be frequently conducted by grassroots health institutions and have high feasibility.

In this study, the co-occurrence of multiple health service needs refers to the situation where an individual requires various health services simultaneously. This is often due to an individual having multiple health issues, necessitating different services to meet their needs. For instance, a person may need to see a doctor, undergo physical therapy, and receive psychological counseling at the same time. We call this the simultaneous need for multiple health services.

### 2.3 Association rules

Association rule is a method for uncovering potentially meaningful relationships in large datasets, typically represented as “A → B” to indicate the relationship between two itemsets (18). The three main metrics used to evaluate association rules are support, confidence, and lift. Support indicates how frequently the association rule appears in the dataset, reflecting its prevalence, and is calculated as: support (A → B) = P(A, B). Confidence represents the conditional probability of B occurring given that A has occurred, measuring the reliability of the rule; higher confidence suggests a greater likelihood of B following A, thus enhancing the rule’s credibility. It is calculated as: confidence (A → B) = P(A, B)/P(A). Lift measures the accuracy of the association rule, calculated as the ratio of confidence to the probability of itemset B, indicating how much A influences B. It is calculated as: lift (A → B) = confidence (A → B)/P(B) = P(A, B)/(P(A)P(B)). Generally, a rule is considered meaningful only when the lift exceeds 1.

The Apriori algorithm is the most widely used algorithm for frequent itemset mining, first proposed by Agrawal et al. (18). It employs an iterative, layer-by-layer search method to mine frequent itemsets. The algorithm first identifies all frequent 1-itemsets (L1), then uses L1 to find frequent 2-itemsets (L2), and continues this process until no frequent k-itemsets can be found. Ultimately, the algorithm extracts association rules from all frequent itemsets that meet a specified confidence threshold, which are the rules of interest to the user. In this study, the Apriori algorithm is used to discover the co-occurrence patterns of multiple health service needs throughout the life course. After several parameter adjustments and trials, it was determined that setting support at 0.1 (10%) and confidence at 0.5 (50%) maximized the number of effective rules. Therefore, this study establishes a minimum support of 1.0% and a minimum confidence of 50%.

### 2.4 Statistical methods

In this study, we utilized EpiData 4.6 for data entry and database creation, and R version 4.3.1 for statistical analysis, with *p* < 0.05 indicating statistical significance. To analyze the relationships and strengths of multiple health service needs across the life course, we employed the arules package in R for data mining using the Apriori algorithm.

## 3 Results

### 3.1 Overall situation of multiple health service needs

The health service need rate among Chinese residents reached 86.76%, with 78.72% of the population having two or more health service needs simultaneously. Among the 12 types of health services, the highest need levels were for health exercise guidance services (17.10%), traditional Chinese medicine health care services (15.53%), and online health information services (14.40%). Overall, the top three multiple health service needs were traditional Chinese medicine consultation services (100.00%), rehabilitation services (99.91%), and mental health services (97.47%), indicating that residents show a greater inclination toward support in traditional Chinese medicine and mental health, reflecting current priorities and trends in the health service sector (Table 1).

**Table 1:** Overall situation of multiple health service needs among Chinese Residents [*n* (%)].

Health service need	*n*	Single	Multiple		Multiple health service needs rate
			2 Types	≥3 Types	
Modern medical care (inpatient and outpatient)	2,791	174 (6.23%)	454 (16.27%)	2,163 (77.50%)	2,617 (93.77%)
Traditional Chinese medicine consultation needs	308	0 (0.00%)	14 (4.55%)	294 (95.45%)	308 (100.00%)
Traditional Chinese medicine health care	5,750	316 (5.50%)	955 (16.61%)	4,479 (77.90%)	5,434 (94.50%)
Health guidance	4,350	269 (6.18%)	690 (15.86%)	3,391 (77.95%)	4,081 (93.82%)
Physical exercise guidance	6,331	584 (9.22%)	1,259 (19.89%)	4,488 (70.89%)	5,747 (90.78%)
Internet medical care and information	5,333	556 (10.43%)	1,278 (23.96%)	3,499 (65.61%)	4,777 (89.57%)
Mental health care	2,451	60 (2.45%)	279 (11.38%)	2,110 (86.09%)	2,389 (97.47%)
Home care	1,212	32 (2.64%)	95 (7.84%)	1,063 (87.71%)	1,158 (95.54%)
Chronic disease management	4,197	295 (7.03%)	656 (15.63%)	3,381 (80.56%)	4,037 (96.19%)
Rehabilitation	2,222	2 (0.09%)	107 (4.82%)	2,113 (95.27%)	2,220 (99.91%)
Child management	1,378	1,254 (91.00%)	93 (6.75%)	31 (2.25%)	124 (9.00%)
Pregnancy health management	702	25 (3.56%)	101 (14.39%)	576 (82.05%)	677 (96.44%)

### 3.2 Visualization of co-occurrence structures

**Figure 1:**
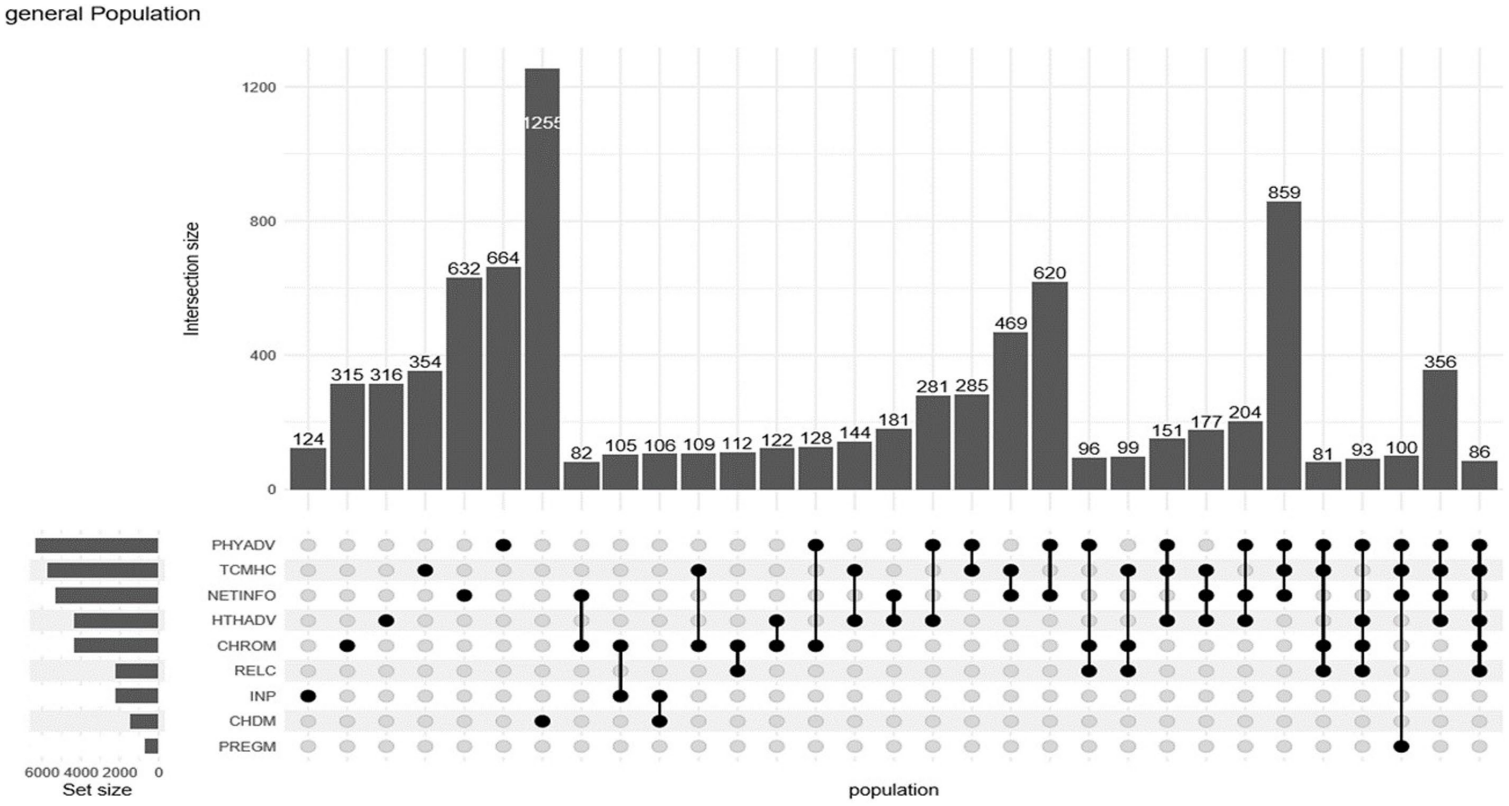
UpSet diagram of co-occurring needs for health services. (1) PHYADV: Physical exercise guidance; (2) TCMHC: Traditional Chinese medicine health care; (3) NETINFO: Internet medical care and information; (4) HTHADV: Health guidance; (5) CHROM: Chronic disease management; (6) RELC: Rehabilitation; (7) INP: Modern medical care (inpatient and outpatient); (8) CHDM: Child management; (9) PREGM: Pregnancy health management.

To gain deeper insights into the distribution of co-occurrence structures among residents, an UpSet plot was created to illustrate the health service needs across the entire sample. For better readability, only the portions representing groups with more than 100 individuals are displayed. The various combinations of needs can be seen in Figure 1. The upper section features a bar chart that corresponds to the number of individual or multiple points (sets) shown in the lower section. The bar chart in the lower left indicates the quantity of corresponding row variables, with each variable representing a specific health service need. Notably, the number of residents with single-item needs is quite low, while the distribution of combined needs appears more varied. The most frequently occurring combination is “exercise guidance needs + internet health information needs + traditional Chinese medicine health care needs,” followed by “exercise guidance needs + internet information needs,” with the third being “internet health information needs + traditional Chinese medicine health care.” Among the combinations not depicted due to lower numbers (under 100), modern medical needs, rehabilitation service needs, and chronic disease management needs occur with higher frequency. Overall, the sample indicates that residents primarily favor preventive health service needs. Although the number of residents experiencing disease (including those ill in the past two weeks and non-acute chronic disease patients) is relatively small, the diversity of their health service needs remains significant.

### 3.3 Co-occurrence rules of multiple needs throughout the lifecourse

**Figure 2:**
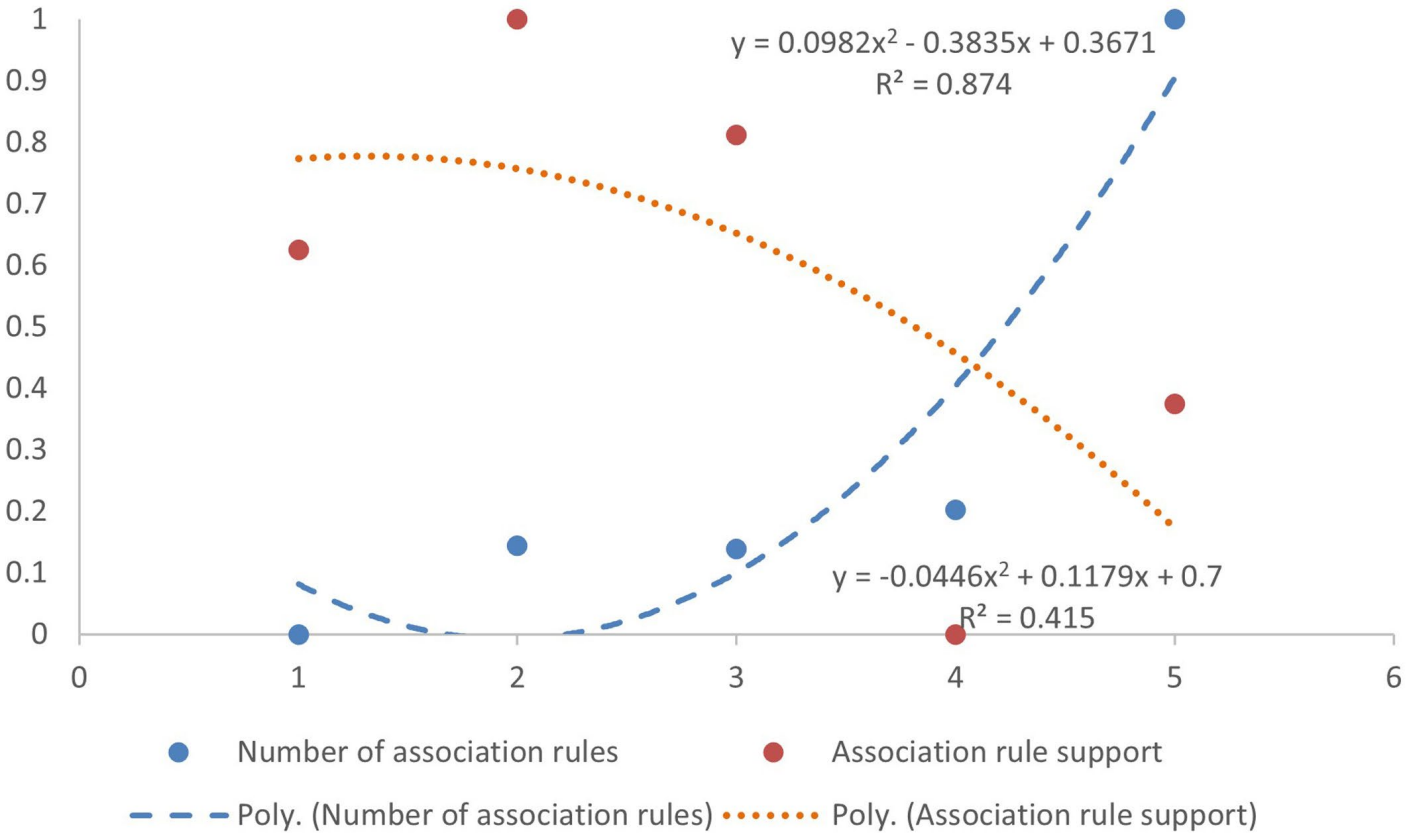
Dimensionless scatter plot of association rules by lifecourse group.

The analysis of the co-occurrence association structure of health service needs reveals that need characteristics throughout the lifecourse do not follow a linear pattern. Specifically, the multi-co-occurrence probability indicated by support exhibits a fluctuating pattern of increase, decrease, and then increase again. Meanwhile, the diversity of the co-occurrence structure, as reflected by the number of rules, shows a trend from low to high. Figure 2 illustrates that the older adult group (x = 5) has the highest number of rules, while its support level is similar to that of the children’s group. This suggests that the health service needs of the older adult are more diverse.

#### 3.3.1 Life preparation stage – infancy

The analysis results of association rules related to children’s medical and preventive health services are presented in Table 2. In the life preparation stage, the analysis identified three association rules for both binary and ternary patterns, with the highest support for the children’s group being below 10%, indicating a low co-occurrence probability. However, when the current or subsequent items include two or more services, the lift significantly increases (lift >13), suggesting that there is a high overall need probability for children needing multiple services. Notably, modern medical care and child management have the highest support, while the combination of modern medical care, traditional Chinese medicine consultation, and child management yields the highest lift at 13.59.

**Table 2:** Results of association rules for children’s health service needs.

Antecedent	Consequent	Support (%)	Confidence (%)	Lift
Modern medical care	Child management	9.34	88.80	1.97
Traditional Chinese medicine consultation	Child management	1.09	86.67	1.96
Traditional Chinese medicine consultation	Modern medical care	1.01	80.00	1.95
Modern medical care, traditional Chinese medicine consultation	Child management	1.84	83.33	13.59
Traditional Chinese medicine consultation	Modern medical care, child management	1.84	66.67	13.59
Modern medical care	Child management, traditional Chinese medicine consultation	1.84	34.29	13.07

#### 3.3.2 Life protection stage – adulthood

In the Life Protection Stage, there are a total of 12 association rules, comprising 7 binary and 5 ternary rules. The top three rules with the highest support include the combinations of Traditional Chinese Medicine (TCM) services with Exercise Guidance, Internet Information with Exercise Guidance, and Internet Information with TCM services. The highest confidence rules are: Psychological Counseling alongside Internet Information and TCM services; Psychological Counseling alongside Internet Information and Exercise Guidance; and Internet Information in combination with TCM services and Exercise Guidance. For further details, refer to Table 3.

**Table 3:** Life protection stage association rules results.

Antecedent	Consequent	Support (%)	Confidence (%)	Lift
Traditional Chinese medicine health care	Physical exercise guidance	21.12	55.55	1.33
Internet medical care and information	Physical exercise guidance	20.34	57.70	1.38
Internet medical care and information	Traditional Chinese medicine health care	19.50	55.30	1.45
Health guidance	Physical exercise guidance	15.62	54.32	1.30
Mental health	Physical exercise guidance	10.20	62.98	1.50
Mental health	Traditional Chinese medicine health care	9.40	58.04	1.53
Mental health	Internet medical care and information	8.67	53.51	1.52
Internet medical care and information, traditional Chinese medicine health care	Physical exercise guidance	12.39	63.55	1.52
Health guidance, traditional Chinese medicine health care	Physical exercise guidance	8.27	57.84	1.38
Mental health, traditional Chinese medicine health care	Physical exercise guidance	6.28	66.81	1.60
Mental health, internet medical care and information	Physical exercise guidance	6.05	69.79	1.67
Mental health, internet medical care and information	Traditional Chinese medicine health care	5.96	68.80	1.81

#### 3.3.3 Life maintenance stage – older adult

The need characteristics of the older adult population aged 65 and above are marked by a scattered distribution, as evidenced by the lower number of association rules and support levels compared to the general population. When the support level is set at 5%, the number of rules generated is similar to that of the general population at a 10% support level. This indicates that the co-occurrence probability of itemsets in the older adult population is lower than that of the general group, suggesting that while there are many potential co-occurrence combinations, their occurrence frequency is low. Consequently, for the life-sustaining population, a minimum support of 5% and a minimum rule confidence of 70% were established. Ultimately, 31 association rules were identified, including 12 binary rules, 17 ternary rules, and 2 quaternary rules.

Within these 31 rules, the right-side itemset comprises only 6 types of health service needs: chronic disease management is involved in 12 rules, traditional Chinese medicine health care in 10 rules, physical exercise guidance and health guidance each in 3 rules, while older adult care and rehabilitation services are included in 2 and 1 rules, respectively. The three association rules with the highest support are: rehabilitation and chronic disease management, home care and chronic disease management, and modern medical care and chronic disease management. The three association rules with the highest confidence are: health guidance with rehabilitation and older adult care; health guidance with modern medical care and traditional Chinese medicine health care; and rehabilitation with chronic disease management. The specific results are detailed in Table 4.

**Table 4:** Association rule results for life maintenance stage.

Antecedent	Consequent	Support (%)	Confidence (%)	Lift
Rehabilitation	Chronic disease management	31.24	99.5	1.55
Home care	Chronic disease management	25.8	70.89	1.10
Modern medical care	Chronic disease management	23.05	79.67	1.24
Home care	Traditional Chinese medicine health care	18.97	52.13	1.19
Home care	Physical exercise guidance	18.34	50.39	1.17
Modern medical care	Traditional Chinese medicine health care	14.86	51.37	1.17
Internet medical care and information	Physical exercise guidance	9.14	55.15	1.31
Mental health	Health guidance	8.98	54.2	1.24
Mental health	Traditional Chinese medicine health care	8.82	58.13	1.33
Internet medical care and information	Traditional Chinese medicine health care	8.69	52.48	1.44
Mental health	Home care	8.69	52.48	1.22
Mental health	Physical exercise guidance	7.24	62.06	1.42
Health guidance, home care	Traditional Chinese medicine health care	12.08	56.09	1.28
Physical exercise guidance, home care	Health guidance	11.82	64.48	1.53
Health guidance, physical exercise guidance	Traditional Chinese medicine health care	11.1	54.0	1.23
Physical exercise guidance, home care	Traditional Chinese medicine health care	10.02	54.66	1.25
Modern medical care, home care	Traditional Chinese medicine health care	7.21	57.43	1.31
Health guidance, modern medical care	Traditional Chinese medicine health care	7.08	60.7	1.44
Modern medical care, home care	Health guidance	6.96	56.27	1.29
Physical exercise guidance, modern medical care	Traditional Chinese medicine health care	5.83	68.14	1.61
Modern medical care, rehabilitation	Chronic disease management	11.26	99.44	1.55
Modern medical care, health guidance	Chronic disease management	10.28	81.86	1.28
Home care, rehabilitation	Chronic disease management	19.1	99.18	1.55
Home care, health guidance	Chronic disease management	15.84	73.57	1.15
Physical exercise guidance, rehabilitation	Chronic disease management	15.33	99.59	1.55
Health guidance, traditional Chinese medicine health care	Chronic disease management	15.33	73.04	1.14
Home care, traditional Chinese medicine health care	Chronic disease management	14.67	77.33	1.20
Health guidance, rehabilitation	Home care	12.77	72.53	1.99
Modern medical care, traditional Chinese medicine health care	Chronic disease management	12.05	81.06	1.26
Home care, traditional Chinese medicine health care, chronic disease management	Rehabilitation	11.98	98.7	1.54
Health guidance, rehabilitation, traditional Chinese medicine health care	Chronic disease management	10.5	99.1	1.54

## 4 Discussion

The findings of this study reveal that 78.72% of residents in China experience multiple health service needs, closely corresponding to the chronic disease prevalence rate of 71.18%. Reports from The Lancet indicate that China’s effective coverage index for health services related to non-communicable diseases is significantly lower than that of developed countries, highlighting the unmet health service needs of Chinese residents (19). The three most pressing needs identified are physical exercise guidance (17.10%), traditional Chinese medicine health care (15.53%), and internet health information services (14.40%). Analysis using association rule algorithms suggests a preference among individuals for health institutions to integrate and package these services together. To improve accessibility and convenience in health services, grassroots health institutions should enhance the integration of health service resources and optimize resource allocation. This can facilitate information sharing and complementary resources between various healthcare and preventive health agencies. Digital platforms should provide traditional Chinese medicine services and exercise guidance, enabling users to easily access TCM knowledge, health assessments, and tailored exercise recommendations (20). Furthermore, leveraging big data and artificial intelligence to analyze user health data can yield targeted interventions, enhancing the effectiveness of TCM health care and increasing user engagement. The development of online courses in TCM and exercise guidance, featuring videos and interactive components, will assist users in grasping TCM theories and practices intuitively. Additionally, establishing an interactive community to encourage users to share their health experiences will promote mutual learning and support (21).

A notable finding from this study is that individuals seeking traditional Chinese medicine (TCM) rarely do so in isolation; they tend to prefer combining TCM consultations with other health services, such as traditional Chinese medicine health care and health guidance. In contrast, children’s health management often leans toward standalone services rather than bundled multiple health services, which reflects the unique healthcare system in China. TCM emphasizes the principle of “harmony and unity,” where various methods are employed collaboratively (22). Recently, national policies have promoted the integration of TCM with Western medicine, encouraging TCM practitioners to incorporate contemporary Western clinical methods into their prescriptions (23). Children’s health management functions as an independent system, where local governments, in collaboration with public healthcare institutions, provide a comprehensive health service package for children from birth. This package includes physical examinations, assessments of growth and development, disease prevention, injury prevention, and dental care (24). Although the health service needs of children are diverse, they are generally encompassed within the basic health service programs mandated by the national government and enforced by local authorities.

Health service needs vary across different life stages. During the life preparation stage, the most sought-after services combine modern medical care, traditional Chinese medicine (TCM) consultations, and child management. In the life protection stage, the primary needs shift to TCM health care, Internet medical information, and health exercise guidance, reflecting a structural consistency and concentration in health service needs. As work-related stress increases, the need for mental health consultations has gradually risen. In the life maintenance stage, the middle-aged and older adult population (ages 45 to 64) displays a richer variety of co-occurring need combinations due to natural aging and the heightened risk of diseases, although the support levels of these rules do not vary significantly. For those aged 65 and older, a marked increase in the need for chronic disease management, modern medical care, and health preservation services arises from declining physical functions and a rising prevalence of chronic illnesses, leading to a greater likelihood of co-occurring needs. The analysis of association rules reveals that the combination of chronic disease management services and rehabilitation services is the most common, with the number of rules for other combinations significantly exceeding those in younger age groups. According to the “long tail theory,” the number of scattered co-occurring combinations, in addition to those concentrated at the “head,” also merits attention. Within the aging context, diverse co-occurrence structures among the older adult—especially those with chronic diseases—are prevalent, and the “long tail” needs of older adults should not be neglected (25). Historically, China’s approach to fulfilling residents’ health service needs has primarily centered on the professional healthcare system, resulting in an incomplete understanding. The “big health” concept promoted by the 20th National Congress emphasizes the need to integrate health considerations into all aspects of public policy development and implementation (26), which inherently requires multiple stakeholders to collaboratively address residents’ diverse health service needs. Only by fully recognizing the implications of this concept can we transform the current hospital-centric service model through institutional arrangements, fostering the connection, integration, and collaboration of health systems. This approach will help establish a shared value consensus and social norms focused on the health needs of residents.

### 4.1 Limitation

This study also has some limitations: Firstly, this is a cross-sectional study. Although it is based on a large sample size survey of primary data, it relies on existing data and cannot account for future changes in health service needs; therefore, policy recommendations must be dynamically adjusted in light of the evolving social environment. Moreover, the study primarily focuses on rural areas in China, which may not fully represent the health service needs of urban residents. Future research could expand the sample scope to include more regions. Additionally, the reliance on association rule algorithms during the analysis may impact the results, as the quality and completeness of the data are crucial. The questionnaire may also be subject to recall bias, with participants potentially forgetting or misremembering certain information, thus affecting the accuracy of their responses. Lastly, while this study emphasizes examining health service need combinations from a life course perspective, future research could enhance the understanding of residents’ health service needs by exploring other dimensions such as gender and socio-economic status, which would provide stronger guidance for practical policies.

## 5 Conclusion

In the study of co-occurrence of multiple health service needs throughout the life course based on association rule algorithms, we discovered significant associations between health service needs at different life stages. The research indicates that the health service need rate among Chinese residents is 86.76%, with 78.72% having two or more needs. The services with the highest need levels are Physical Exercise Guidance (17.10%), Traditional Chinese Medicine Health Care (15.53%), and Internet Medical Care and Information (14.40%). The top three multiple needs are Traditional Chinese Medicine Consultation (100.00%), Rehabilitation Services (99.91%), and Mental Health Services (97.47%), with the highest co-occurrence frequency found in the combination of “Exercise Guidance + Internet Health Information + Traditional Chinese Medicine Health Care.” The characteristics of these needs fluctuate across life stages. In the life preparation stage, the main needs are for modern medical care and child management; in the life protection stage, the focus is on traditional Chinese medicine health care and physical exercise guidance; while in the life maintenance stage, there is a notable increase in the need for chronic disease Management and Rehabilitation Services. Therefore, we recommend constructing a comprehensive health service system that spans the entire life course, ensuring individuals receive personalized services while emphasizing accessibility and equity to achieve widespread access to quality health services.

## Data Availability

The raw data supporting the conclusions of this article will be made available by the authors, without undue reservation.
